# The effect of population size for pathogen transmission on prediction of COVID-19 spread

**DOI:** 10.1038/s41598-021-97578-9

**Published:** 2021-09-09

**Authors:** Xuqi Zhang, Haiqi Liu, Hanning Tang, Mei Zhang, Xuedong Yuan, Xiaojing Shen

**Affiliations:** 1 School of Mathematics, Sichuan University, Chengdu, 610064 Sichuan China; 2 School of Computer Science, Sichuan University, Chengdu, 610064 Sichuan China

**Keywords:** Statistics, Applied mathematics

## Abstract

Extreme public health interventions play a critical role in mitigating the local and global prevalence and pandemic potential. Here, we use population size for pathogen transmission to measure the intensity of public health interventions, which is a key characteristic variable for nowcasting and forecasting of COVID-19. By formulating a hidden Markov dynamic system and using nonlinear filtering theory, we have developed a stochastic epidemic dynamic model under public health interventions. The model parameters and states are estimated in time from internationally available public data by combining an unscented filter and an interacting multiple model filter. Moreover, we consider the computability of the population size and provide its selection criterion. With applications to COVID-19, we estimate the mean of the effective reproductive number of China and the rest of the globe except China (GEC) to be 2.4626 (95% CI: 2.4142–2.5111) and 3.0979 (95% CI: 3.0968–3.0990), respectively. The prediction results show the effectiveness of the stochastic epidemic dynamic model with nonlinear filtering. The hidden Markov dynamic system with nonlinear filtering can be used to make analysis, nowcasting and forecasting for other contagious diseases in the future since it helps to understand the mechanism of disease transmission and to estimate the population size for pathogen transmission and the number of hidden infections, which is a valid tool for decision-making by policy makers for epidemic control.

## Introduction

In December 2019, a new type of coronavirus pneumonia named COVID-19 spread very rapidly in the city of Wuhan, China^1^. It swept the globe in the next few months. This outbreak of COVID-19 is now present in at least 180 countries and the virus has infected over 60,000,000 people with more than 1,500,000 deaths as of December 8, 2020. This new coronavirus has been considered to be a true global health emergency. The World Health Organization (WHO) announced the COVID-19 to be a global pandemic on March 11, 2020 local time in Geneva. Faced with the severe epidemic trend of COVID-19, many countries have declared a state of emergency and have instituted drastic actions and interventions to stop the spread of the virus. For example, China implemented unprecedented intervention strategies on January 23, 2020 to prevent further outbreak of COVID-19. These policies have included large-scale quarantine, strict controls on travel and extensive monitoring of suspected cases which have made a huge difference in controlling the spread of the virus. Nevertheless, the outbreak is still continuing and having a devastating impact in Europe, USA and many other countries. Many scientific research groups in the world including those in China, Europe, and USA are working independently or together by sharing their results and experiences, including analyzing the mechanism of disease transmission^2–4^ and identifying optimal control measures^5–7^ for countries where the outbreak is in its early stages. In addition, the prevention and control experience of the 2003 SARS epidemic^8–10^, the worldwide 2009 H1N1 influenza pandemic^11^, and the 2012 MERS-CoV^12^ are also being used for COVID-19.

**Figure 1:**
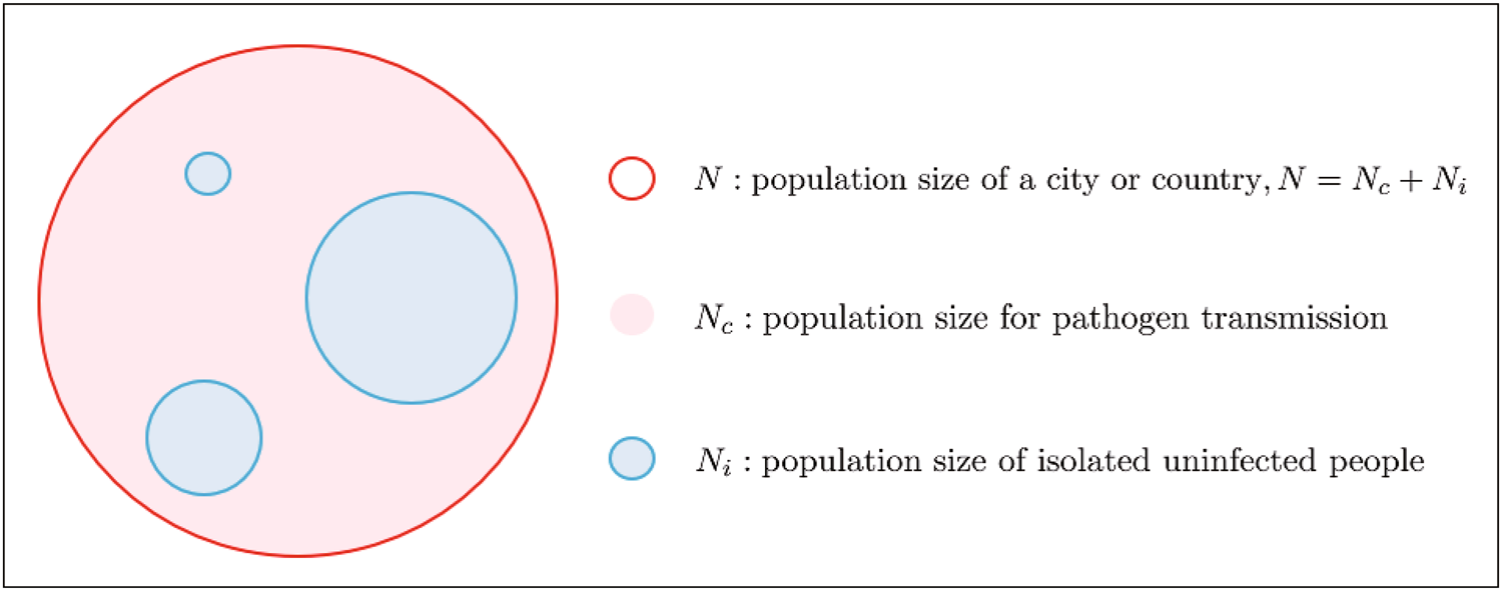
The total population and its division.

Mathematical modeling and prediction tools are extremely important tools for decision-making by policy makers for epidemic control. The classical SIR, SEIR and GLEAM models are extensively used^13–19^ for comprehending the mechanism of disease transmission, its spread and forecasting. These approaches have become remarkably successful in free infection and transmission patterns and predicting the temporal evolution of ongoing epidemics. However, under strict control measures, the abundance of different, often mutually incompatible, forecasting results, suggest that we still lack a fundamental understanding of the key factors of infection and transmission dynamics. Recently, several epidemiological studies^20–22^ show that the population size is a crucial transmissibility factor, which could increase or decrease the transmissibility risk. Note that the evolution of an epidemic is a process of gradual spread, i.e., the number of people in contact with the infected persons increase gradually. In particular, when strict control measures are adopted to reduce the risk of the transmission, the population size of pathogen transmission may decrease to a new population size of a small control region. Therefore, the population size should be carefully considered in an epidemic model and it is not appropriate to keep in a fixed population size of the city or country for making accurate predictions. Here, we assume that there exists a closed subsystem in a city or country when the uninfected people are in home quarantine or some cities are locked down. The population size of the subsystem is considered as the population size for pathogen transmission, which is usually much smaller than the total population of a city or county when deploying intervention strategies. The total population is divided into the population for pathogen transmission and the isolated uninfected population (see Fig. 1).

In this paper, we use the population size $N_c$ for pathogen transmission to measure the intensity of public health interventions, discuss the identifiability of the population size $N_c$ and propose an intuitive and efficient criterion to select the unknown population size. Furthermore, we concentrate on three specific aims: (i) Modeling: develop mathematical differential equation dynamic models that account for the random variations in pathogen, society, and public health intervention variables etc.; (ii) Nowcasting: estimate the states and parameters as a function of time through nonlinear filtering with publicly available international data of COVID-19. Moreover, we analyze the key factor, the number of latent infections, which determines the size of the newly confirmed infections in the next seven to fourteen days in terms of the incubation period^4,23^; (iii) Forecasting: predict local and global spread trends (e.g. scales, peaks and confidence intervals) of the infections under different control intensities, i.e., different population sizes for pathogen transmission.

Our contributions mainly lie in three aspects. First of all, we develop a stochastic epidemic dynamic model under public health interventions. By formulating a hidden Markov dynamic system, the model parameters and states are estimated in time from publicly available international data by combining an unscented filter and an interacting multiple model filter. Secondly, we consider the computability of the population size for pathogen transmission and provide a selection criterion. Here, we use it to measure the intensity of public health interventions, which is a key characteristic variable for estimation and prediction of the epidemic. Finally, we provide comprehensive nowcasting and forecasting results of some countries, which show the effectiveness of the stochastic epidemic dynamic model with nonlinear filtering. The mean of the effective reproductive number of China was estimated to be 2.4626 (95% CI: 2.4142–2.5111), which is consistent with what was reported in the literature.

## Methods

### Mathematical epidemic modeling

It is well recognized that random effects are often unavoidable in reality and a population is often affected by random disturbances. Due to factors such as climate change, population density, public health intervention, and medical care capability, the parameters of epidemiological models may also experience random changes during transmission. Some researchers have devoted themselves to studying stochastic epidemic models through stochastic differential equation models and methods^16^. In this section, we introduce a stochastic susceptible-exposed-infectious-recovered (SEIR) model for the free propagation stage of the epidemic, where random variables and processes are included in the epidemiological process model to characterize the uncertainty associated with the propagation of the epidemic. Moreover, we develop a stochastic SEIR model under public health interventions.

#### The stochastic SEIR model

Based on the classical SEIR model proposed by Aron and Schwartz^15^ that is deterministic in nature. In order to account for system uncertainties, we modify the SEIR model by introducing random noises and random parameters in the SEIR model as follows:

1 $$\begin{aligned} \frac{\mathrm {d} \it{S(t)}}{{\mathrm {d}} t}=-\alpha (t) \cdot \frac{S(t) \cdot I(t)}{N}+w_{S}\it{(t)}, \end{aligned}$$

2 $$\begin{aligned} \frac{\mathrm {d} \it{E(t)}}{\mathrm {d} \it{t}}=\alpha (t) \cdot \frac{S(t) \cdot I(t)}{N}-\beta (t)\cdot E(t)+w_{E}(t), \end{aligned}$$

3 $$\begin{aligned} \frac{\mathrm {d} \it{I(t)}}{\mathrm {d} \it{t}}=\beta (t)\cdot E(t) -(\gamma ^1(t)+\gamma ^2(t)) \cdot I(t)+w_{I}(t), \end{aligned}$$

4 $$\begin{aligned} \frac{\mathrm {d} \it{R(t)}}{\mathrm {d} \it{t}}=\gamma ^1(t) \cdot I(t)+w_{R}(t), \end{aligned}$$

5 $$\begin{aligned} \frac{\mathrm {d} \it{D(t)}}{\mathrm {d} \it{t}}=\gamma ^2(t) \cdot I(t)+w_{D}(t), \end{aligned}$$

6 $$\begin{aligned} \frac{\mathrm {d}\mathbf{p}\it{(t)}}{\mathrm {d}\it{t}}=\mathbf{w}_{\mathbf{p}}(t), \end{aligned}$$

where *S*(*t*), *E*(*t*), *I*(*t*), *R*(*t*) and *D*(*t*) are the number of the susceptible, exposed, infectious, recovered and disease-caused death cases at time *t*, respectively. The parameter vector $\mathbf{p}(t)=(\alpha (t), \beta (t), \gamma ^1(t), \gamma ^2(t))^T$ includes the mean contact rate, transfer rate from exposed to infective, recovery rate and disease-caused mortality. Since these model parameters may experience random changes in the spreading process due to different climate changes, population densities, public health interventions, and medical cares in different regions, we model the uncertainty associated with the parameters as Brownian motions, which could be explained by a random walk with a small noise due to the fact that the parameters represent average random characteristics of a large number of members of the total population^24^, and $\mathbf{w}_{\mathbf{p}}(t)=(w_{\alpha }(t), w_{\beta }(t),w_{\gamma ^1}(t),w_{\gamma ^2}(t))^T$ is a white noise process with spectral density $\mathbf{Q}_{\mathbf{p}}(t)$. Moreover, the basic reproductive number $R_0= \alpha /(\gamma ^1+\gamma ^2)$ is defined as the average number of secondary infections caused by an infected individual in a completely susceptible population, and the effective reproduction number $R_e$ is defined as the average number of secondary infections caused by an infected individual in the population after there is some immunity or interventions have been put in place^25,26^. Besides, the birth rate, natural death rate and other uncertainties are considered as a zero mean white noise process $\mathbf{w}_s(t)$ with spectral density $\mathbf{Q}_{\mathbf{w}_s}(t)$, which is defined as follows:

$$\begin{aligned} \mathbf{w}_s(t)=\left( w_{S}(t),w_{E}(t),w_{I}(t),w_{R}(t),w_{D}(t)\right) ^T. \end{aligned}$$

Since the epidemic happens in a closed community, we further assumed that the total population size remains constant in a short epidemic period, which is commonly used in stochastic epidemic models^16,27,28^, and

7 $$\begin{aligned} S(t) + E(t) + I(t) +R(t) +D(t)= N. \end{aligned}$$

The stochastic model (1)–(6) is suitable for the free propagation of epidemic without control measures. It also characterizes the uncertainties of parameters and states in the propagation process.

#### The stochastic SEIR model under public health interventions

When there are public health interventions and the infections are isolated, we extend the stochastic SEIR model as follows:

8 $$\begin{aligned} \frac{\mathrm {d} \it{S_c(t)}}{\mathrm {d} \it{t}}=-\alpha _c(t) \cdot \frac{S_c(t) \cdot E_c(t)}{N_c}+w_{S_c}(t), \end{aligned}$$

9 $$\begin{aligned} \frac{\mathrm {d} \it{E_c(t)}}{\mathrm {d} \it{t}}=\alpha _c(t) \cdot \frac{S_c(t) \cdot E_c(t)}{N_c}-\beta _c(t)\cdot E_c(t) +w_{E_c}(t), \end{aligned}$$

10 $$\begin{aligned} \frac{\mathrm {d} \it{I_c(t)}}{\mathrm {d} \it{t}}=\beta _c(t)\cdot E_c(t)-(\gamma _c^1(t)+\gamma _c^2(t)) \cdot I_c(t)+w_{I_c}(t), \end{aligned}$$

11 $$\begin{aligned} \frac{\mathrm {d} \it{R_c(t)}}{\mathrm {d} \it{t}}=\gamma _c^1(t) \cdot I_c(t)+w_{R_c}(t), \end{aligned}$$

12 $$\begin{aligned} \frac{\mathrm {d} \it{D_c(t)}}{\mathrm {d} \it{t}}=\gamma _c^2(t) \cdot I_c(t)+w_{D_c}(t), \end{aligned}$$

13 $$\begin{aligned} \frac{\mathrm {d}\mathbf{p}\it{_c(t)}}{\mathrm {d}\it{t}}=\mathbf{w}_{\mathbf{p}_c}(t), \end{aligned}$$

where $S_c$ and $N_c$ are the susceptible and the population size under public health interventions, respectively. $E_c(t)$ and $I_c(t)$ are redefined as the number of latent infections, and confirmed infections at time *t*, respectively. The reason is that the confirmed infections can be observed each day and it is convenient to estimate the model parameters and forecast the number of confirmed and latent infections by nonlinear filtering methods. Moreover, the infectious *I*(*t*) in (1) are replaced by $E_c(t)$ in (8), which means that the susceptible are not infected by isolated infections. The parameters $\mathbf{w}_{s_c}(t)=(w_{S_c}(t), w_{E_c}(t), w_{I_c}(t),w_{R_c}(t),w_{D_c}(t))^T$, $\mathbf{w}_{\mathbf{p}_c}(t)$ and $\mathbf{p}_c(t)$ are similarly defined as that in the stochastic model (1)–(6).

It is worth noting that under public health interventions, we assume that there exists a closed subsystem in a city or country when the uninfected people are in home quarantine or some cities are locked down. The population size of the subsystem is considered as the population size for pathogen transmission $N_c$, which is usually much smaller than the urban or national population when deploying intervention strategies. The total population is divided into the population for pathogen transmission and the isolated uninfected population (see Fig. 1). In fact, the population size $N_c$ plays a key role in nowcasting and forecasting epidemic trends and measures of control intensity. Herein, we assumed that $N_c$ satisfies (7) as well. This paper aims to analyze the data of COVID-19 epidemic when the population size $N_c$ is unknown.

For simplicity, the subscript *c* of $N_c, S_c , E_c, I_c, R_c, D_c, \mathbf{p}_{c}, \mathbf{w}_{\mathbf{p}_c}$ and $\mathbf{w}_{s_c}$ is omitted when there is no confusion. Specifically, the population size *N* refers to the population size for pathogen transmission in the rest of this paper.

### Nowcasting and forecasting methods

In this section, we provide the observation model for the stochastic SEIR dynamic system (8)–(13) as well as methods for nowcasting, forecasting and the selection criterion of the population size for pathogen transmission *N*.

#### Continuous-discrete dynamic system

It is worth to note that the stochastic SEIR model (8)–(13) satisfies the linear equality constraint (7) as well, which can be used to reduce the system model parameterization. By substituting $S=N-E-I-R-D$ in the model (8)–(13), we have the following equivalent reduction model:

14 $$\begin{aligned} \frac{\mathrm {d} \it{E(t)}}{\mathrm {d} \it{t}}=-\alpha (t)\cdot E(t)\cdot \frac{E(t)+I(t)+R(t)+D(t)}{N}+(\alpha (t)-\beta (t))\cdot E(t)+w_{E}(t), \end{aligned}$$

15 $$\begin{aligned} \frac{\mathrm {d} \it{I(t)}}{\mathrm {d} \it{t}}=\beta (t) \cdot E(t)-(\gamma ^1(t)+\gamma ^2(t)) \cdot I(t)+w_{I}(t), \end{aligned}$$

16 $$\begin{aligned} \frac{\mathrm {d} \it{R(t)}}{\mathrm {d} \it{t}}=\gamma ^1(t) \cdot I(t)+w_{R}(t), \end{aligned}$$

17 $$\begin{aligned} \frac{\mathrm {d} \it{D(t)}}{\mathrm {d} \it{t}}=\gamma ^2(t) \cdot I(t)+w_{D}(t), \end{aligned}$$

18 $$\begin{aligned} \frac{\mathrm {d}\mathbf{p}\it{(t)}}{\mathrm {d}\it{t}}=\mathbf{w}_{\mathbf{p}}(t), \end{aligned}$$

where *S*(*t*) is determined from the equality constraint (7). Moreover, we denote the state vector and process noise as

$$\begin{aligned} \mathbf{x}(t)&=(E(t), I(t), R(t), D(t),\mathbf{p}(t)^T)^T,\\ \mathbf{w}(t)&=(w_{E}(t), w_{I}(t),w_{R}(t),w_{D}(t),\mathbf{w}_{\mathbf{p}}(t)^T)^T, \end{aligned}$$

where $\mathbf{w}(t)$ is a white noise process with spectral density $\mathbf{Q}(t)$. On the basis of the state $\mathbf{x}(t)$ defined in the model (14)–(18) and the prevalence data that can be obtained, we model the observation equation as follows:

19 $$\begin{aligned} {\mathbf {z}}_{k}\triangleq&(M_k^c,R_k^c,D_k^c)^T= \mathbf{H}\mathbf{x}(t_k)+\mathbf{v}_{k}, \end{aligned}$$

where $\mathbf{z}_k$ is the measurement of the system state $\mathbf{x}(t_k)$, which includes the number of the cumulative confirmed infections $M_k^c$, cumulative recovered cases $R^c_k$ and cumulative disease-caused death cases $D^c_k$ at time $t_k$. The statistical error associated with the measurements is modeled as a Gaussian white measurement noise $\mathbf{v}_{k}$ with a small covariance $\mathbf{R}_{k}$, and $\mathbf{H}$ is a linear measurement matrix defined as follows:

$$\begin{aligned} \mathbf{H}&=\left( \begin{array}{cccccccc} 0 &{} 1 &{} 1 &{} 1 &{} 0 &{} 0 &{} 0 &{} 0\\ 0 &{} 0 &{} 1 &{} 0 &{} 0 &{} 0 &{} 0 &{} 0\\ 0 &{} 0 &{} 0 &{} 1 &{} 0 &{} 0 &{} 0 &{} 0\\ \end{array} \right) . \end{aligned}$$

As a consequence, we have the following unconstrained continuous-discrete dynamic system:

20 $$\begin{aligned} \frac{\mathrm {d}\mathbf{x}(t)}{\mathrm {d}t}&=f(\mathbf{x}(t),\mathbf{w}(t)), \end{aligned}$$

21 $$\begin{aligned} \mathbf{z}_{k}&=\mathbf{H}\mathbf{x}(t_k)+\mathbf{v}_{k}, \end{aligned}$$

where $f(\cdot )$ is a simplified expression of the drift function corresponding to (14)–(18). The process noise $\mathbf{w}(t)$, measurement noise $\mathbf{v}_k$, and initial state are assumed to be mutually independent.

#### Nowcasting and forecasting

For the nonlinear system (20)–(21), the optimal Bayesian state estimation is usually intractable. There are several strategies to approximate the optimal estimation, such as the unscented Kalman filter (UKF)^29,30^, particle filter and cubature Kalman filter^31,32^ etc. For the system under consideration, we employ the continuous-discrete UKF^29,30^ to estimate the posterior expectation of the random state and parameters recursively by the daily data $\mathbf{z}_k$. Specific steps of the continuous-discrete UKF are shown in supplementary information, where the continuous state model is implemented by the fourth order Runge-Kutta method^33^.

##### Remark 1

Since there are sudden changes in the states and parameters, e.g., the scheme to designate the confirmed infections in China was revised on February 12, 2020 so that the number of cumulative confirmed infections had a drastic increase of 15152 persons^34^. The transfer rate $\beta (t)$ should be modeled differently before and after February 12, 2020. Moreover, the classical interacting multiple model (IMM) filter in maneuvering target tracking^35^ can be used to enhance the stability of the algorithm and improve the performance of the nowcasting and forecasting methods.

Furthermore, the prediction probability density of the state ${\mathbf {x}}(t)$ is derived by the model prediction step of UKF through the model (14)–(18) with posterior parameters. In this paper, we adopt the expectation of the predicted state as the prediction result. Let $\mathbf{m}_{i+j|i}=(I_{i+j|i}, R_{i+j|i}, D_{i+j|i})^T$ denote the vector consisting of the expectation of the confirmed infections, recovered cases and disease-caused death cases predicted from time $t_i$ to $t_{i+j}$, where $M^c_{i+j|i}$ $=I_{i+j|i}+R_{i+j|i}+D_{i+j|i}$ represents the expectation of the cumulative confirmed infections predicted from time $t_i$ to $t_{i+j}$, and $\sigma _{i+j|i}$ is the standard deviation of the error of $M^c_{i+j|i}$. Then, the approximate 95% confidence intervals (CIs) are calculated as follows:

$$\begin{aligned} (M^c_{i+j|i}-2\sigma _{i+j|i},\ M^c_{i+j|i}+2\sigma _{i+j|i}). \end{aligned}$$

Based on the proposed stochastic SEIR model under public health interventions (14)–(18), the nowcasting and forecasting methods described above, the local and global spread trends of the epidemic are analyzed under public health interventions and different control intensities, respectively.

#### Selection criterion of the population size *N*

When the epidemic spreads freely in a city, the urban population can be chosen as the population size *N*. However, under public health interventions, the value of *N* should be selected carefully, and we use *N* to measure the intensity of interventions. In this section, we investigate the identifiability of population size and provide the selection criterion.

##### Lemma 1

*Considering the deterministic SEIR model (i.e., neglecting the random noises and randomness of the deterministic parameters in* (1)–(5)), *if the states* *E*, *I*, *R*, *D* *and model parameters have a perturbation* ${\mathbf {O}}(\frac{1}{N})$ *which is infinitesimal of the same order of* $\frac{1}{N}$, *and the population size* $N=S+E+I+R+D$ *is large enough, then the forecast results in finite time interval derived by Runge-Kutta integration are with perturbation* ${\mathbf {O}}(\frac{1}{N})$ *as well.*

##### Proof

See supplementary information. $\square$

Note that the *E*, *I*, *R*, *D* are integer valued in practical applications, based on Lemma 1, we conclude that the prediction results are the same almost everywhere when the population size *N* is large enough. Moreover, we have the same results on forecasting and filtering as follows:

##### Proposition 1

*If the current state estimation and covariance at time* $t_k$ *have a perturbation* ${\mathbf {O}}(\frac{1}{N})$ *which has a compatible dimension and the population size* $N=S+E+I+R+D$ *is large enough, then the state prediction* ${\hat{\mathbf{x}}}(t)$ *and covariance* $\mathbf{P}(t)$ *in finite time derived by the model prediction step of the continuous-discrete UKF via Runge-Kutta integration are with perturbations* ${\mathbf {O}}(\frac{1}{N})$ *as well.*

##### Proof

See supplementary information. $\square$

##### Proposition 2

*If the state estimation and covariance at time*$t_k$*are with perturbations*${\mathbf {O}}(\frac{1}{N})$*which has a compatible dimension and the population size*$N=S+E+I+R+D$*is large enough, then the state update of*${\hat{\mathbf{x}}}(t)$*and*$\mathbf{P}(t)$*in finite time through continuous-discrete UKF via Runge-Kutta integration are still with perturbations*${\mathbf {O}}(\frac{1}{N})$.

##### Proof

See supplementary information. $\square$

Based on the recursive method, Propositions 1-2 show that *N* cannot be estimated since the state estimates are the same almost everywhere for different *N* when they are large. Thus, it is unnecessary to consider a very large *N*, especially under strict control measures or early on in an outbreak. On the other hand, it is not difficult to show that *N* can be estimated when it is not too large since $\frac{E\left( E+I+R+D\right) }{N}$ is not infinitesimal (see the analysis in supplementary information). Moreover, we provide a selection criterion for the value of *N* by solving the following optimization problem, i.e., we select the population size by minimizing the average relative prediction error of the cumulative confirmed infections in the next *J* time steps:

22 $$\begin{aligned} \begin{aligned} \mathop {\min }_{N}\,\frac{1}{J}\sum _{j=1}^{J} e_j(N) \\ \text {s.t.}\,N\le N_u, \end{aligned} \end{aligned}$$

where *J* is a constant, e.g., 7 days. *N* is the variable over which optimization is to be carried out, $N_u$ may be half of the regional population or other values based on prior data, which depends on the intensity of the control measures etc., and the $e_j(N)$ is calculated as follows:

23 $$\begin{aligned} e_j(N)=\frac{1}{k-j}\sum _{i=1}^{k-j}\left| \frac{M^c_{i+j|i}(N)-M^c_{i+j}}{M^c_{i+j}}\right| , \end{aligned}$$

where $M^c_{i+j}$ and $M^c_{i+j|i}(N)$ are the observations of the cumulative confirmed infections and the prediction of the cumulative confirmed infections based on the population size *N* through time $t_i$ to $t_{i+j}$ for $j=1, \ldots , J$, respectively.

### Results

Based on the proposed stochastic SEIR model under public health interventions (14)–(18) and the nowcasting and forecasting methods, the spread trends of COVID-19 epidemic in several countries such as China, India, Iran, Russia and USA are analyzed under different control intensities (i.e., different population size for pathogen transmission *N*). All the data are collected from daily reports of National Health Commission of the People’s Republic of China (NHC) and Tencent News real-time epidemic tracking^36,37^, including the numbers of cumulative confirmed infections, recovered cases and disease-caused death cases.

In order to make predictions of epidemic spread, we need to estimate the parameters $\alpha (t), \beta (t), \gamma ^1(t), \gamma ^2(t)$ accurately. By implementing continuous-discrete UKF, the posterior expectation of the parameters in the model (14)–(18) are derived. $\alpha (t)$ is the contact rate representing the average rate of transmission for the susceptible to latent infections, and we choose the initial value of $\alpha (t)$ between 0.1-0.2^6^. $\beta (t)$ is the average transfer rate from exposed to infective and $\frac{1}{\beta (t)}$ represents the incubation period. Because the incubation period of the COVID-19 has been reported to be between 0 to 24 days^4^, we choose 7–14 days, i.e., the initial value of $\beta (t)$ is between $\frac{1}{7}$ and $\frac{1}{14}$. And $\gamma ^1(t)$ is the average rate of recovery (i.e., $\gamma ^1(t) = \frac{1}{D}$, where *D* is the average duration of the infection), we choose the average duration of the infection between 10 days to 20 days, i.e., the initial value of $\gamma ^1$ is between 0.05 and 0.1. Here we use the disease-caused mortality 0.1$\%$-3$\%$^38^. Additionally, the initial values of *I*, *R*, *D* are set as the number of confirmed cases, recovery cases and disease-caused death cases reported on the day of the first confirmed infections was found, respectively.

#### The selection of the population size *N* and estimation of the effective reproductive number

**Figure 2:**
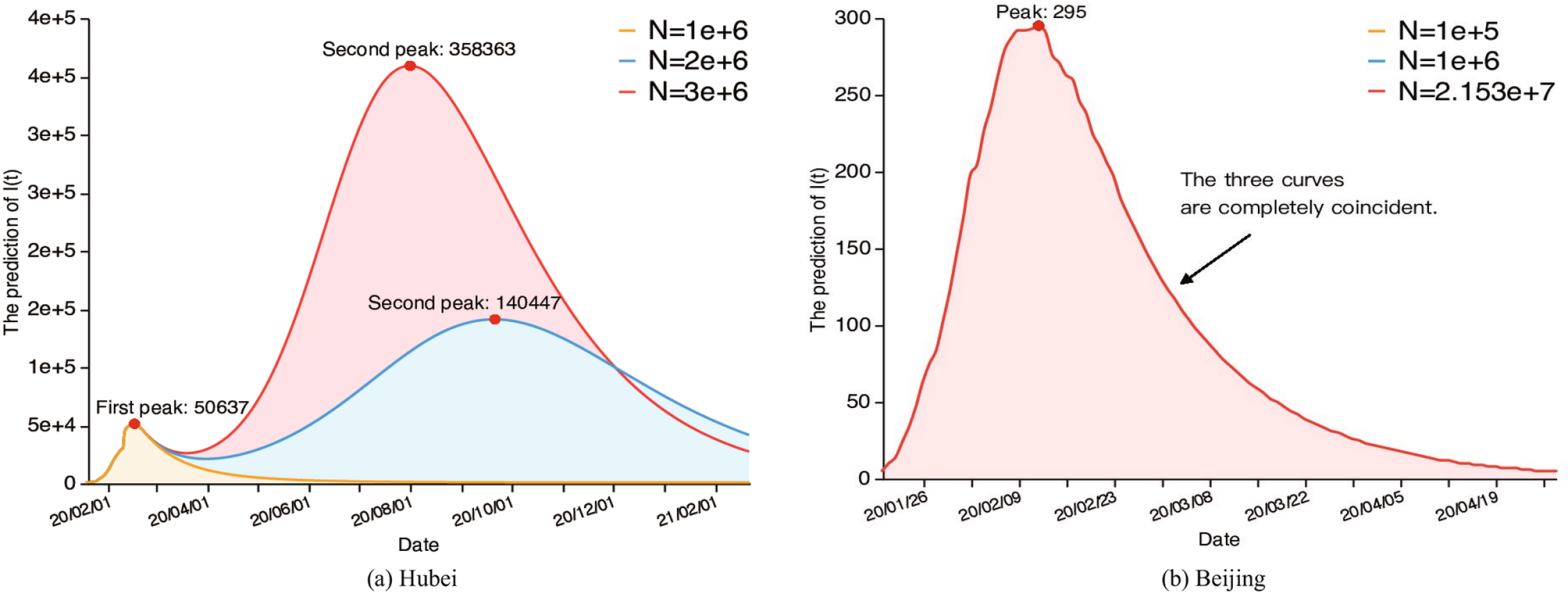
The forecasting of the epidemic trend of *I*(*t*) with different population sizes.

Figure 2a shows the prediction trends of the confirmed infections *I*(*t*) based on the data through January 20 to February 24 of Hubei Province, China under different intensities of public health interventions, i.e, maintaining the control intensity and the population size $N=1,000,000$, relaxing the control intensity and the population sizes $N=2,000,000$, and $N=3,000,000$, respectively. The results indicate that relaxing the Hubei quarantine would lead to a second epidemic peak, and the larger the population size *N*, the higher the peak, which will lead to the selection of different control measures. It also illustrates the effect of the population size *N*, due to the prediction trends of confirmed infections in hospitals are completely different when using different population sizes in the model. Thus, the selection of the population size for pathogen transmission is a key factor for the forecasting of the epidemic and *N* is identifiable for this case. Figure 2b shows the prediction for Beijing City. It indicates that when *N* is greater than 100,000, the predicted values are the same, i.e, *N* is unidentifiable. This is consistent with Propositions 1-2. In fact, early on in the epidemic, Beijing took very strict control measures so that the effective reproductive number $R_e$ is very small, the small number of infections cannot spread to a larger population.

Therefore, for global prediction, it is required to select the population size carefully. Through the selection criterion (22) and setting $J=7$, we select the population size for pathogen transmission of China and the globe except China (GEC) based on the prevalence data of COVID-19 epidemic^36,37^. The selected population size *N* of China is about 1,220,000 derived by using data from January 21, 2020 to March 18, 2020 when there were no local newly confirmed cases^36^. And the selected population size *N* of GEC is about 224,000,000 derived by using data from January 21, 2020 to December 8, 2020. It indicates that both the selected population sizes of China and GEC are far less than the total population.

**Figure 3:**
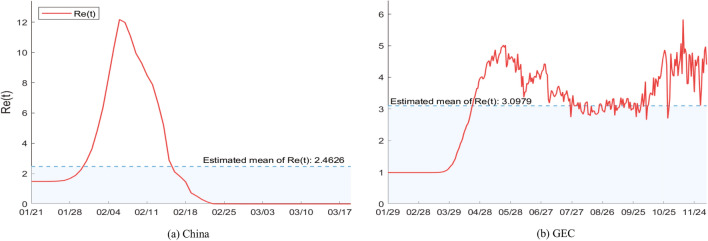
Estimation results for the effective reproductive number.

Figure 3a shows the estimation of the effective reproductive number $R_e$ of China from January 21, 2020 to March 18, 2020 using the selected population size $N=1,220,000$. It shows that the effective reproductive number $R_e$ increases monotonically early on in the outbreak, then monotonically decreases with a sharp infection point. This is consistent with the policy decision in which Chinese government implemented unprecedented public health interventions to prevent the spread of the epidemic and the incubation period is about 7–14 days^4^. The reason for monotonic increase in the early stages of the epidemic may be that, in a limited local city, the density of infections increases as a result of the infections increasing exponentially. It results in a quick growth of the contact rate $\alpha (t)$. The recovery rate is very low due to the recovery cycle. The reason for monotonic decrease is that the public health interventions (e.g. scales of quarantine, strict controls on travel, hand hygiene, and use of face masks etc.^39^) significantly decrease the contact rate $\alpha (t)$ and the recovery rate gradually increases, which results in a monotonic decrease of the effective reproductive number $R_e$. In Fig. 3b, it can be seen that the curve of the effective reproductive number $R_e$ of GEC reached its first peak later than that of China, due to the fact that the coronavirus has been detected in many countries since February, 2020 and the response measures of these countries were later than China. Due to the implementation of strict public health interventions in European countries and USA in March to contain the pandemic, the spread of the virus has been curbed to a certain extent so that the effective reproductive number $R_e$ of GEC declined. However, the effective reproductive number $R_e$ had a second peak. The reasons may be that the isolation control measures have been relaxed in many countries, and the epidemic in some other countries such as Brazil, India, Russia became serious from May. The current effective reproductive number $R_e$ of GEC is still high and fluctuates around 3.

We also present the estimated mean values and 95% CI of the key epidemiological parameters of GEC and China in Table 1. The mean of the effective reproductive number of COVID-19 in China is 2.4626 (95% CI: 2.4142–2.5111). This finding aligns with other recent estimates of the effective reproductive number for this time period^5,40–44^.

**Table 1:** The estimated mean and 95% CI of parameters.

Parameters	GEC	China
Contact rate $\alpha$	0.1055 $\left( 0.0927, 0.1182\right)$	0.0714 $\left( 0, 0.2260\right)$
Transfer rate $\beta$	0.0937 $\left( 0.0683, 0.1191\right)$	0.0554 $\left( 0, 0.1841\right)$
Remove rate $r^1+r^2$	0.0453 $\left( 0.0418, 0.0488\right)$	0.0584 $\left( 0, 0.1221\right)$
Effective reproductive number $R_e$	3.0979 $\left( 3.0968, 3.0990\right)$	2.4626 $\left( 2.4142, 2.5111\right)$

Moreover, we select the population size of several countries such as India, Iran, Russia and USA based on the prevalence data of COVID-19 epidemic^37^ from the date of the first confirmed infection to December 8, 2020 under the same selection criterion. Table 2 gives the selected values of *N* and national populations^45^ of several major epidemic affected countries. Here, we restate that *N* is the population size for pathogen transmission. The total national population is divided into the population for pathogen transmission and the isolated uninfected population. It shows that the selected *N* under public health interventions are far less than the national population.

**Table 2:** The selected *N* and national population.

Country	India	Iran	Russia	USA
Population	1,298,041,000	82,083,918	143,436,145	327,167,434
Selected *N*	54,000,000	4,300,000	6,600,000	67,000,000

**Figure 4:**
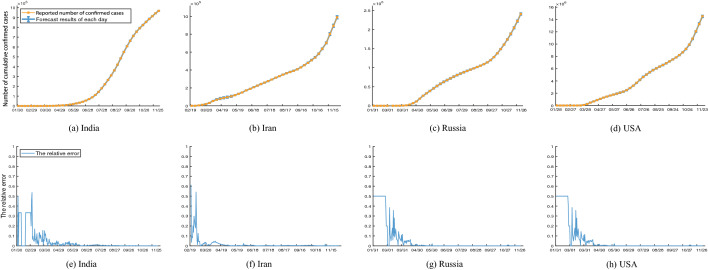
Daily prediction of one time step and 95% CI derived by the model (14)–(18) though continuous-discrete UKF.

To validate the selected population sizes *N*, Fig. 4 shows the reported confirmed infections from the date of the first confirmed case to December 8, 2020, daily prediction of one time step, the corresponding 95% CIs and the relative error between daily prediction of one time step and reported confirmed infections for several major epidemic affected countries, respectively. Due to the large amount of data, in order to show the results more clearly, we only draw the data with an interval of seven days in Fig. 4 and the following pictures. It indicates that the 95% CIs can cover the true confirmed infections, even in the outbreak phase (see Fig. 4a–d). Figure 4e–h shows that all of the relative error curves converge after several days and have a small error, which show that our model, selected population size *N* and the parameters estimated through nonlinear filtering can accurately predict the real data in a short period, qualifying it for making nowcasting and forecasting. Furthermore, more prediction results of 1-7 days are updated and available on https://github.com/SCU-Bigdata/2019-nCoV-Forecast in time since February 7, 2020.

#### The nowcasting and forecasting results

**Figure 5:**
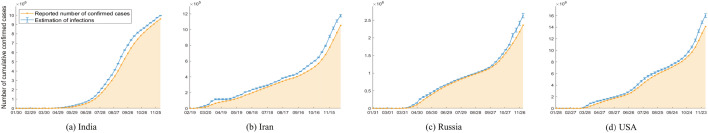
The reported number of cumulative confirmed infections and the estimate of the total number of infections.

Under the selected population size *N*, the number of latent infections *E*(*t*) can be estimated in time again by UKF. Figure 5 shows the reported cumulative confirmed infections, the estimate of the actual infections and corresponding 95% CIs, where the difference between the two lines represents *E*(*t*). It indicates that the situation in USA is the most serious one and it has 2,295,026 latent infections on December 8, 2020. The latent infections account for a large fraction 12.99% of the total number of infections and based on the incubation period of 7–14 days^4^, we infer that the pandemic will continue for a long time in USA, unless more stringent public health interventions are taken to reduce the spread of COVID-19. Similar results are anticipated for India, Russia with 327,922 and 273,583 latent infections, respectively. Compared with the other three countries, Iran has relatively few hidden infections, about 121,817 cases.

**Figure 6:**
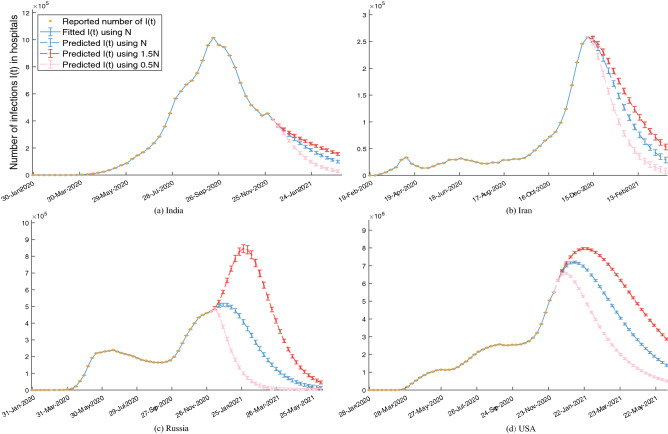
The impact of different control intensities implemented by changing the population size on the *I*(*t*).

Figure 6 shows that the reported number of confirmed infections in hospitals of some major countries, the fitted number using the selected population size *N* through nonlinear filtering, and the forecast results including the number, corresponding 95% CIs, peak and arrival time of the infections in hospitals under different control measures by changing the population size for pathogen transmission and for fixed contact rate, e.g., 1) maintaining the selected population size *N* for pathogen transmission, 2) relaxing the control intensity and the population size *N* has a 50% increase, 3) strengthening the control intensity and the population size *N* has a 50% reduction. As shown in Fig. 6, the fitted values are accurate and the 95% CIs can cover reported number of confirmed infections in hospitals, even if the reported number of *I*(*t*) has many fluctuations. We find that except India and Iran, by decreasing of the population size, i.e., strict control measures, the peaks would have a decrease, especially in USA and Russia, and the time of peaks would arrive earlier. It is worth noting that this is different from the results of Wu et al.^5^. The reason is that Wu et al.^5^ assume that the population size is fixed and strengthen the control intensity by reducing the contact rate $\alpha (t)$. Thus, it is not contradictory. In this experiment, there are no effects on the peak of different population sizes in India and Iran, but the decline rates of the infections in hospitals are different. If the population size continues to grow, there could be a second peak. The prediction results in Fig. 6 indicate that the use of different population sizes *N* to make prediction will have different trends and peaks. Therefore, it is necessary to take public health intervention measures to avoid the spread of COVID-19 to a larger subsystem, in other words, to avoid an increase in the population size *N* for pathogen transmission.

## Discussion

Nowcasting and forecasting of epidemics play a vital role in predicting geographic disease spread as well as case counts so as to better inform public health interventions when outbreaks occur^46^. In this study, we developed a stochastic SEIR model under public health interventions, the nonlinear filtering and prediction methods and selection criterion for the population size for pathogen transmission, which could be valuable to national and international agencies for public health situation perception and design of intervention strategies. Our technical contributions are made with a backdrop of the COVID-19 pandemic that has engulfed the world. We infer that USA has the most serious situation as it has 2,295,026 latent infections as of December 8, 2020. India and Russia are with 327,922 and 273,583 latent infections, respectively. Based on the present trajectory, the latent infections may cause further outbreaks in the case of weak public health interventions.

Therefore, it is essential to strengthen control intensity such as large-scale quarantine, strict controls on travel and extensive monitoring of suspected cases to reduce the contact rate and the population size of the susceptible. Here we regard the population size *N* for pathogen transmission as one of the measures of the control intensity, which is conducive to assess the effectiveness of public health interventions. Moreover, it is valuable to obtain the more accurate number of the latent infections, which is helpful to design control strategies (e.g., scales of quarantine, strict controls on travel, contact isolation, hand hygiene, and use of face masks) in the future. However, on the population size *N*, we selected it by minimizing the prediction error based only on the current data, which may be prone to overfitting of the data. One possible improvement approach is to include the prediction covariance in the selection criterion. Besides, the stochastic model (14)–(18) ignored the impact of the imported cases, which are considered as random noises. If the airline transportation data can be obtained, by combining the networked dynamic metapopulation model^14,19,47^ and selection of the local population sizes in time, more accurate prediction methods and results for the local and global trend may be obtained. We believe that it is extremely important to predict further spread of COVID-19 globally and evaluate the effectiveness of different mitigation strategies and to try to mitigate the impact of this pandemic on the entire global population.

## Supplementary Information

Supplementary Information.

## Data Availability

The author confirms that the data used in this paper are freely available to public: https://github.com/SCU-Bigdata/2019-nCoV-Forecast/tree/master/Collect$\_$data.
