# Supplementary material for: The effect of population size for pathogen transmission on prediction of COVID-19 spread
Source: Sci Rep. 2021 Sep 9;11:18024. doi: 10.1038/s41598-021-97578-9 (PMC8429718; doi:10.1038/s41598-021-97578-9)
Supplement: Supplementary file 1 — Supplementary Information. [file 41598_2021_97578_MOESM1_ESM.pdf]

The Effect of Population Size for Pathogen Transmission on Prediction of COVID-19 Spread: Supplementary Information

Xuqi Zhang[1], Haiqi Liu[1,*], Hanning Tang[1], Mei Zhang[1], Xuedong Yuan[2], and Xiaojing Shen[1]

[1]School of Mathematics, Sichuan University, Chengdu, Sichuan, 610064, China
[2]School of Computer Science, Sichuan University, Chengdu, Sichuan, 610064, China
[*]Corresponding author.@email:411566818@qq.com

The Continuous-discrete UKF

The main steps of the continuous-discrete UKF[1,2] are summarized as follows. We define a matrix operation

$$\mathbf{Y} = g(\mathbf{X}),$$

where $\mathbf{X} \in \mathbb{R}^{n\times p}$ and $\mathbf{Y} \in \mathbb{R}^{n\times q}$, and the i-th column $\mathbf{Y}_i$ of the matrix $\mathbf{Y}$ is calculated from $g(\mathbf{X}_i)$.

- Model prediction step: the state and covariance prediction $\hat{\mathbf{x}}(t_{k|k-1})$, $\mathbf{P}(t_{k|k-1})$ are implemented by Runge-Kutta integration from initial conditions $\hat{\mathbf{x}}(t_{k-1})$ and $\mathbf{P}(t_{k-1})$:

$$\frac{\mathrm{d}\mathbf{x}(t)}{\mathrm{d}t} = f(\mathbf{X}(t),t)w_m, \tag{1}$$

$$\frac{\mathrm{d}\mathbf{P}(t)}{\mathrm{d}t} = \mathbf{X}(t)\mathbf{W}f^T(\mathbf{X}(t),t) + f(\mathbf{X}(t),t)\mathbf{W}\mathbf{X}^T(t) + \mathbf{Q}(t), \tag{2}$$

where $\mathbf{x}(t)$ and $\mathbf{P}(t)$ are the n-dimensional column vector and $n\times n$ dimensional positive semidefinite matrix, respectively. f is the state transition function, the $(2n+1)\times(2n+1)$ dimensional sigma points matrix $\mathbf{X}(t)$, the weight vector w_m and the weight matrix $\mathbf{W}$ are defined as follows:

$$\mathbf{X}(t) = \begin{pmatrix}\mathbf{x}(t) & \cdots & \mathbf{x}(t)\end{pmatrix} + \sqrt{n+\lambda}\begin{pmatrix}\mathbf{0}_{n\times 1} & \sqrt{\mathbf{P}(t)} & -\sqrt{\mathbf{P}(t)}\end{pmatrix}, \tag{3}$$

$$w_m = \begin{pmatrix}W_0^{(m)} & \dots & W_{2n}^{(m)}\end{pmatrix}^T, \tag{4}$$

$$\mathbf{W} = \left(\mathbf{I}_{2n+1} - \begin{pmatrix}w_m & \dots & w_m\end{pmatrix}\right)\times \mathrm{diag}\begin{pmatrix}W_0^{(c)} & \dots & W_{2n}^{(c)}\end{pmatrix}\times\left(\mathbf{I}_{2n+1} - \begin{pmatrix}w_m & \dots & w_m\end{pmatrix}\right)^T, \tag{5}$$

$$W_0^{(m)} = \frac{\lambda}{(n+\lambda)}, \tag{6}$$

$$W_0^{(c)} = \frac{\lambda}{(n+\lambda)+(1-\theta^2+\kappa)}, \tag{7}$$

$$W_i^{(m)} = \frac{1}{2(n+\lambda)}, \quad i=1,\dots,2n, \tag{8}$$

$$W_i^{(c)} = \frac{1}{2(n+\lambda)}, \quad i=1,\dots,2n, \tag{9}$$

where $\sqrt{\mathbf{P}(t)}$ is the matrix square root of $\mathbf{P}(t)$. $\mathbf{0}_{n\times 1}$, $\mathbf{I}_{2n+1}$ are the n-dimensional zero column vector, $(2n+1)$-dimensional identity matrix, respectively. θ, κ, μ are positive constant parameters and $\lambda = \theta^2(n+\mu) - n$.

- Measurement update step: Combining the new measurement $\mathbf{z}_k$ at time t_k with the state prediction $\hat{\mathbf{x}}(t_{k|k-1})$ and covariance $\mathbf{P}(t_{k|k-1})$ obtained in the forecast step, the state is updated via information gain matrix $\mathbf{K}_k$,

$$\hat{\mathbf{x}}(t_k) = \hat{\mathbf{x}}(t_{k|k-1}) + \mathbf{K}_k \left(\mathbf{z}_k - \hat{\mathbf{z}}_{k|k-1}\right), \tag{10}$$
$$\mathbf{P}(t_k) = \mathbf{P}(t_{k|k-1}) - \mathbf{K}_k \operatorname{Cov}\left(\hat{\mathbf{z}}_{k|k-1}\right) \mathbf{K}_k^T, \tag{11}$$

where the gain matrix $\mathbf{K}_k$, and the measurement covariance $\operatorname{Cov}\left(\hat{\mathbf{z}}_{k|k-1}\right)$ are computed as follows:

$$\hat{\mathbf{X}}(t_{k|k-1}) = \begin{pmatrix}\hat{\mathbf{x}}(t_{k|k-1}) & \cdots & \hat{\mathbf{x}}(t_{k|k-1})\end{pmatrix} + \sqrt{n+\lambda}\cdot\begin{pmatrix}\mathbf{0}_{n\times 1} & \sqrt{\mathbf{P}(t_{k|k-1})} & -\sqrt{\mathbf{P}(t_{k|k-1})}\end{pmatrix}, \tag{12}$$
$$\hat{\mathbf{Z}}_{k|k-1} = \mathbf{H}\hat{\mathbf{X}}(t_{k|k-1}), \tag{13}$$
$$\hat{\mathbf{z}}_{k|k-1} = \hat{\mathbf{Z}}_{k|k-1} w_m, \tag{14}$$
$$\operatorname{Cov}(\hat{\mathbf{z}}_{k|k-1}) = \hat{\mathbf{Z}}_{k|k-1}\mathbf{W}\hat{\mathbf{Z}}_{k|k-1}^T + \mathbf{R}_k, \tag{15}$$
$$\operatorname{Cov}(\hat{\mathbf{x}}(t_{k|k-1}), \hat{\mathbf{z}}_{k|k-1}) = \hat{\mathbf{X}}(t_{k|k-1})\mathbf{W}\hat{\mathbf{Z}}_{k|k-1}^T, \tag{16}$$
$$\mathbf{K}_k = \operatorname{Cov}(\hat{\mathbf{x}}(t_{k|k-1}), \hat{\mathbf{z}}_{k|k-1})\operatorname{Cov}^{-1}(\hat{\mathbf{z}}_{k|k-1}). \tag{17}$$

More details can be seen in the articles [1,2].

Proof of Lemma 1

Since $N = S + E + I + R + D$, the deterministic SEIR model can be simplified as follows:

$$\frac{\mathrm{d}E(t)}{\mathrm{d}t} = \alpha \cdot \frac{(N - E(t) - I(t) - R(t) - D(t)) \cdot I(t)}{N} - \beta \cdot E(t), \tag{18}$$
$$\frac{\mathrm{d}I(t)}{\mathrm{d}t} = \beta \cdot E(t) - (\gamma^1 + \gamma^2) \cdot I(t), \tag{19}$$
$$\frac{\mathrm{d}R(t)}{\mathrm{d}t} = \gamma^1 \cdot I(t), \tag{20}$$
$$\frac{\mathrm{d}D(t)}{\mathrm{d}t} = \gamma^2 \cdot I(t), \tag{21}$$

where the parameters α, β, γ^1, γ^2 are constants, and $S = N - E - I - R - D$ holds for all time. Denote the state with a perturbation as

$$\tilde{\mathbf{x}}(t_k) = \mathbf{x}(t_k) + \mathbf{O}(\frac{1}{N}),$$

where $\mathbf{x}(t_k) = \left(E(t_k), I(t_k), R(t_k), D(t_k), \alpha, \beta, \gamma^1, \gamma^2\right)^T$ represents the true state at time t_k. We use the simplified expression $f_d(\mathbf{x}(t), t)$ as the drift function in differential equations (18)-(21). Herein, we adopt the fourth order Runge-Kutta method to derive the state $\tilde{\mathbf{x}}(t_m)$ at time t_m by using initial condition $\tilde{\mathbf{x}}(t_k)$.

$$\tilde{\mathbf{x}}(t_m) = \tilde{\mathbf{x}}(t_k) + \frac{h}{6}(\tilde{k}_1 + 2\tilde{k}_2 + 2\tilde{k}_3 + \tilde{k}_4), \tag{22}$$
$$\tilde{k}_1 = f_d(\tilde{\mathbf{x}}(t_k), t_k), \tag{23}$$
$$\tilde{k}_2 = f_d(\tilde{\mathbf{x}}(t_k) + \frac{h}{2}\tilde{k}_1, t_k + \frac{h}{2}), \tag{24}$$
$$\tilde{k}_3 = f_d(\tilde{\mathbf{x}}(t_k) + \frac{h}{2}\tilde{k}_2, t_k + \frac{h}{2}), \tag{25}$$
$$\tilde{k}_4 = f_d(\tilde{\mathbf{x}}(t_k) + h\tilde{k}_3, t_k + h), \tag{26}$$

where h is the time interval $t_m - t_k$. By ignoring the higher order terms than $\mathbf{O}(\frac{1}{N})$ generated in the nonlinear transformation of f_d, we have

$$\begin{aligned}\tilde{k}_1 &= f_d(\tilde{\mathbf{x}}(t_k), t_k) \\ &= f_1(\mathbf{x}(t_k), t_k) + f_N(\mathbf{x}(t_k), t_k),\end{aligned} \tag{27}$$

where $f_d(\tilde{\mathbf{x}}(t_k),t_k)$, $f_N(\mathbf{x}(t_k),t_k)$ and $f_1(\mathbf{x}(t_k),t_k)$ are

$$f_d(\tilde{\mathbf{x}}(t_k),t_k) = \begin{pmatrix} a_d \\ b_d \\ (\gamma^1+\mathbf{O}(\frac{1}{N}))\cdot(I(t_k)+\mathbf{O}(\frac{1}{N})) \\ (\gamma^2+\mathbf{O}(\frac{1}{N}))\cdot(I(t_k)+\mathbf{O}(\frac{1}{N})) \end{pmatrix},$$

$$f_N(\mathbf{x}(t_k),t_k) = \begin{pmatrix} a_N \\ b_N \\ \gamma^1\cdot\mathbf{O}(\frac{1}{N})+I(t_k)\cdot\mathbf{O}(\frac{1}{N}) \\ \gamma^2\cdot\mathbf{O}(\frac{1}{N})+I(t_k)\cdot\mathbf{O}(\frac{1}{N}) \end{pmatrix},$$

$$f_1(\mathbf{x}(t_k),t_k) = \begin{pmatrix} \alpha\cdot I(t_k)-\beta\cdot E(t_k) \\ \beta\cdot E(t_k)-(\gamma^1+\gamma^2)\cdot I(t_k) \\ \gamma^1\cdot I(t_k) \\ \gamma^2\cdot I(t_k) \end{pmatrix},$$

$$\begin{aligned} a_d &= -(\beta+\mathbf{O}(\frac{1}{N}))\cdot(E(t_k)+\mathbf{O}(\frac{1}{N}))+(N-E(t_k)-I(t_k)-R(t_k)-D(t_k)-\mathbf{O}(\frac{1}{N}))\cdot\frac{(\alpha+\mathbf{O}(\frac{1}{N}))\cdot(I(t_k)+\mathbf{O}(\frac{1}{N}))}{N}, \\ b_d &= -(\gamma^1+\gamma^2+\mathbf{O}(\frac{1}{N}))\cdot(I(t_k)+\mathbf{O}(\frac{1}{N}))+(\beta+\mathbf{O}(\frac{1}{N}))\cdot(E(t_k)+\mathbf{O}(\frac{1}{N})), \\ a_N &= -\alpha\cdot(E(t)+I(t)+R(t)+D(t))\cdot I(t)\cdot\mathbf{O}(\frac{1}{N})+\alpha\cdot\mathbf{O}(\frac{1}{N})+I(t_k)\cdot\mathbf{O}(\frac{1}{N})-\beta\cdot\mathbf{O}(\frac{1}{N})-E(t_k)\cdot\mathbf{O}(\frac{1}{N}), \\ b_N &= \beta\cdot\mathbf{O}(\frac{1}{N})+E(t_k)\cdot\mathbf{O}(\frac{1}{N})-(\gamma^1+\gamma^2)\cdot\mathbf{O}(\frac{1}{N})-I(t_k)\cdot\mathbf{O}(\frac{1}{N}). \end{aligned}$$

Similarly, the k_1 calculated by the initial value $\mathbf{x}(t_k)$ without the permutation $\mathbf{O}(\frac{1}{N})$ is

$$\begin{aligned} k_1 &= f_d(\mathbf{x}(t_k),t_k) \\ &= \overline{f_N}(\mathbf{x}(t_k),t_k)+f_1(\mathbf{x}(t_k),t_k), \end{aligned}$$

where $\overline{f_N}(\mathbf{x}(t_k),t_k)$ is

$$\begin{aligned} \overline{f_N}(\mathbf{x}(t_k),t_k) &= (\overline{a_N},0,0,0)^T, \\ \overline{a_N} &= -\alpha\cdot(E(t)+I(t)+R(t)+D(t))\cdot I(t)\cdot\mathbf{O}(\frac{1}{N}). \end{aligned}$$

Comparing $\tilde{k}_1$ and k_1, we conclude that $\tilde{k}_1 = k_1+\mathbf{O}(\frac{1}{N})$. Similarly from the calculation of (24)-(26), it can be concluded that $\tilde{k}_i = k_i+\mathbf{O}(\frac{1}{N}), i=2,3,4$ as well. By (22), we have

$$\tilde{\mathbf{x}}(t_m) = \mathbf{x}(t_m)+\mathbf{O}(\frac{1}{N}).$$

Proof of Proposition 1

Considering the model prediction step, the Runge-Kutta integration is implemented for the differential equations (1)-(2) to derive the state prediction and covariance. Without loss of generality, we assume that the state and covariance at time t_k are with a perturbation $\mathbf{O}(\frac{1}{N})$,

$$\begin{aligned} \tilde{\mathbf{x}}(t_k) &= \hat{\mathbf{x}}(t_k)+\mathbf{O}(\frac{1}{N}), \\ \tilde{\mathbf{P}}(t_k) &= \mathbf{P}(t_k)+\mathbf{O}(\frac{1}{N}), \end{aligned}$$

where $\hat{\mathbf{x}}(t_k)$, $\mathbf{P}(t_k)$ are the state estimation and covariance at time t_k. By the continuous-discrete UKF, the set of sigma points is chosen via (3),

$$\hat{\mathbf{X}}(t_k) = (\hat{\mathbf{x}}(t_k) \quad \cdots \quad \hat{\mathbf{x}}(t_k))+\sqrt{n+\lambda}\begin{pmatrix}\mathbf{0} & \sqrt{\mathbf{P}(t_k)} & -\sqrt{\mathbf{P}(t_k)}\end{pmatrix}, \tag{28}$$

$$\tilde{\mathbf{X}}(t_k) = (\tilde{\mathbf{x}}(t_k) \quad \cdots \quad \tilde{\mathbf{x}}(t_k))+\sqrt{n+\lambda}\begin{pmatrix}\mathbf{0} & \sqrt{\tilde{\mathbf{P}}(t_k)} & -\sqrt{\tilde{\mathbf{P}}(t_k)}\end{pmatrix}, \tag{29}$$

where $\hat{\mathbf{X}}(t_k)$ and $\tilde{\mathbf{X}}(t_k)$ are derived by the state and covariance without perturbations and with perturbations, respectively.

In light of the first order perturbation bound of Cholesky decomposition[3], we have:

$$\frac{\|\sqrt{\tilde{\mathbf{P}}(t_k)}-\sqrt{\mathbf{P}(t_k)}\|_F}{\|\sqrt{\mathbf{P}(t_k)}\|} \leq \frac{\operatorname{cond}(\mathbf{P}(t_k))}{\sqrt{2}} \frac{\|\tilde{\mathbf{P}}(t_k)-\mathbf{P}(t_k)\|_F}{\|\mathbf{P}(t_k)\|}, \tag{30}$$

where $\|\cdot\|_F$ is the Frobenius norm, and $\operatorname{cond}(\cdot)$ represents the condition number. Since $\mathbf{P}(t_k)$ is a constant matrix and $\tilde{\mathbf{P}}(t_k)-\mathbf{P}(t_k)=\mathbf{O}(\frac{1}{N})$, we have

$$\sqrt{\tilde{\mathbf{P}}(t_k)} = \sqrt{\mathbf{P}(t_k)} + \mathbf{O}(\frac{1}{N}).$$

Rewriting (29), we obtain that

$$\tilde{\mathbf{X}}(t_k) = \hat{\mathbf{X}}(t_k) + \mathbf{O}(\frac{1}{N}), \tag{31}$$

namely, the sigma points of $\tilde{\mathbf{x}}(t_k)$ are also with a perturbation $\mathbf{O}(\frac{1}{N})$ compared with the sigma points of $\hat{\mathbf{x}}(t_k)$.

Thus, we adopt the fourth-order Runge-Kutta method to calculate the state and covariance prediction (1)-(2). By Lemma 1, we have that the state and covariance prediction $\tilde{\mathbf{x}}(t_{k+1|k})$, $\tilde{\mathbf{P}}(t_{k+1|k})$ based on $\tilde{\mathbf{x}}(t_k), \tilde{\mathbf{P}}(t_k)$ are still with a perturbation $\mathbf{O}(\frac{1}{N})$ comparing with the state and covariance $\hat{\mathbf{x}}(t_{k+1|k})$, $\mathbf{P}(t_{k+1|k})$ predicted by $\hat{\mathbf{x}}(t_k)$ and $\mathbf{P}(t_k)$.

Proof of Proposition 2

Denote the state estimation and covariance with perturbations as follows:

$$\begin{aligned}
\tilde{\mathbf{x}}(t_k) &= \hat{\mathbf{x}}(t_k) + \mathbf{O}(\frac{1}{N}), \\
\tilde{\mathbf{P}}(t_k) &= \mathbf{P}(t_k) + \mathbf{O}(\frac{1}{N}),
\end{aligned}$$

By Proposition 1, we know that the state and covariance prediction of $\tilde{\mathbf{x}}(t_k), \tilde{\mathbf{P}}(t_k)$ are still with a perturbation, i.e.,

$$\begin{aligned}
\tilde{\mathbf{x}}(t_{k+1|k}) &= \hat{\mathbf{x}}(t_{k+1|k}) + \mathbf{O}(\frac{1}{N}), \\
\tilde{\mathbf{P}}(t_{k+1|k}) &= \mathbf{P}(t_{k+1|k}) + \mathbf{O}(\frac{1}{N}),
\end{aligned}$$

then the state prediction and covariance $\hat{\mathbf{x}}(t_{k+1|k}), \mathbf{P}(t_{k+1|k})$ without perturbation are updated by measurement $\mathbf{z}_{k+1}$ through (10)-(11), i.e.,

$$\begin{aligned}
\hat{\mathbf{x}}(t_{k+1}) &= \hat{\mathbf{x}}(t_{k+1|k}) + \mathbf{K}_{k+1}\left(\mathbf{z}_{k+1} - \hat{\mathbf{z}}_{k+1|k}\right), \\
\mathbf{P}(t_{k+1}) &= \mathbf{P}(t_{k+1|k}) - \mathbf{K}_{k+1} \operatorname{Cov}\left(\hat{\mathbf{z}}_{k+1|k}\right) \mathbf{K}_{k+1}^T,
\end{aligned}$$

where $\hat{\mathbf{z}}_{k+1|k}, \operatorname{Cov}\left(\hat{\mathbf{z}}_{k+1|k}\right), \mathbf{K}_{k+1}$ are calculated by (12)-(17).

Besides, for the state prediction and covariance $\tilde{\mathbf{x}}(t_{k+1|k})$, $\tilde{\mathbf{P}}(t_{k+1|k})$ with a perturbation, similarly as (31), the sigma points of $\tilde{\mathbf{x}}(t_{k+1|k})$ are with perturbations, i.e.,

$$\tilde{\mathbf{X}}(t_{k+1|k}) = \hat{\mathbf{X}}(t_{k+1|k}) + \mathbf{O}(\frac{1}{N}),$$

by the observation model and equations (13)-(14), we have

$$\begin{aligned}
\tilde{\mathbf{Z}}_{k+1|k} &= \mathbf{H}\tilde{\mathbf{X}}(t_{k+1|k}) \\
&= \hat{\mathbf{Z}}_{k+1|k} + \mathbf{O}(\frac{1}{N}) \\
\tilde{\mathbf{z}}_{k+1|k} &= \mathbf{H}\hat{\mathbf{X}}(t_{k+1|k})w_m + \mathbf{O}(\frac{1}{N}), \\
&= \hat{\mathbf{z}}_{k+1|k} + \mathbf{O}(\frac{1}{N})
\end{aligned}$$

namely, the observations of $\tilde{\mathbf{X}}(t_{k+1|k})$ are still with a perturbation $\mathbf{O}(\frac{1}{N})$ comparing to that of $\hat{\mathbf{X}}(t_{k+1|k})$. The innovation covariance and cross covariance are calculated by (15) and (16),

$$\begin{aligned}
\mathrm{Cov}\left(\tilde{\mathbf{z}}_{k+1|k}\right) &= \tilde{\mathbf{Z}}_{k+1|k}\mathbf{W}\tilde{\mathbf{Z}}_{k+1|k}^T + \mathbf{R}_{k+1} \\
&= \mathrm{Cov}\left(\hat{\mathbf{z}}_{k+1|k}\right) + \mathbf{O}(\frac{1}{N}), \\
\mathrm{Cov}\left(\tilde{\mathbf{x}}(t_{k+1|k}), \tilde{\mathbf{z}}_{k+1|k}\right) &= \tilde{\mathbf{X}}(t_{k+1|k})\mathbf{W}\tilde{\mathbf{Z}}_{k+1|k}^T \\
&= \mathrm{Cov}\left(\hat{\mathbf{x}}(t_{k+1|k}), \hat{\mathbf{z}}_{k+1|k}\right) + \mathbf{O}(\frac{1}{N}),
\end{aligned}$$

and the inverse of matrix with perturbations has following property[4]:

$$\left\|B^{-1} - A^{-1}\right\|_F \leqslant \mu \left\|A^{-1}\right\|_2 \left\|B^{-1}\right\|_2 \|B - A\|_F ,$$

where μ is a constant, $\|\cdot\|_2$ and $\|\cdot\|_F$ represent the Euclidean norm and Frobenius norm, respectively. If we denote that $B = \mathrm{Cov}\left(\tilde{\mathbf{z}}_{k+1|k}\right)$, $A = \mathrm{Cov}\left(\hat{\mathbf{z}}_{k+1|k}\right)$, then we have

$$\left\|\mathrm{Cov}\left(\tilde{\mathbf{z}}_{k+1|k}\right)^{-1} - \mathrm{Cov}\left(\hat{\mathbf{z}}_{k+1|k}\right)^{-1}\right\|_F \leqslant \mu \cdot \left\|\mathbf{O}(\frac{1}{N})\right\|_F \cdot \left\|\mathrm{Cov}\left(\hat{\mathbf{z}}_{k+1|k}\right)^{-1}\right\|_2 \cdot \left\|\mathrm{Cov}\left(\tilde{\mathbf{z}}_{k+1|k}\right)^{-1}\right\|_2 ,$$

Thus,

$$\mathrm{Cov}^{-1}\left(\tilde{\mathbf{z}}_{k+1|k}\right) = \mathrm{Cov}\left(\hat{\mathbf{z}}_{k+1|k}\right)^{-1} + \mathbf{O}(\frac{1}{N}),$$

and the gain matrix $\tilde{\mathbf{K}}_{k+1}$ is

$$\begin{aligned}
\tilde{\mathbf{K}}_{k+1} &= \mathrm{Cov}\left(\tilde{\mathbf{x}}(t_{k+1|k}), \tilde{\mathbf{z}}_{k+1|k}\right) \mathrm{Cov}^{-1}\left(\tilde{\mathbf{z}}_{k+1|k}\right) \\
&= \mathbf{K}_{k+1} + \mathbf{O}(\frac{1}{N}).
\end{aligned}$$

Moreover, the state prediction and covariance are updated by measurement $\mathbf{z}_{k+1}$ as follows:

$$\begin{aligned}
\tilde{\mathbf{x}}(t_{k+1}) &= \tilde{\mathbf{x}}(t_{k+1|k}) + \tilde{\mathbf{K}}_{k+1}\left(\mathbf{z}_{k+1} - \tilde{\mathbf{z}}_{k+1|k}\right) \\
&= \hat{\mathbf{x}}(t_{k+1}) + \mathbf{O}(\frac{1}{N}), \\
\tilde{\mathbf{P}}(t_{k+1}) &= \tilde{\mathbf{P}}(t_{k+1|k}) - \tilde{\mathbf{K}}_{k+1}\mathrm{Cov}^{-1}\left(\tilde{\mathbf{z}}_{k+1|k}\right)\tilde{\mathbf{K}}_{k+1}^T \\
&= \mathbf{P}(t_{k+1}) + \mathbf{O}(\frac{1}{N}).
\end{aligned}$$

Therefore, the state estimation and covariance at time t_{k+1} are with perturbations $\mathbf{O}(\frac{1}{N})$ as well.

References

1. Julier, S. J. & Uhlmann, J. K. Unscented filtering and nonlinear estimation. *Proc. IEEE* **92**, 401–422 (2004).

2. Sarkka, S. On unscented Kalman filtering for state estimation of continuous-time nonlinear systems. *IEEE Transactions on Autom. Control.* **52**, 1631–1641 (2007).

3. Bhatia, R. Matrix factorizations and their perturbations. *Linear Algebr. Its Appl.* **197**, 245–276 (1994).

4. Wedin, P.-Å. Perturbation theory for pseudo-inverses. *BIT Numer. Math.* **13**, 217–232 (1973).
